# Percutaneous Tibial Nerve Stimulation in the Treatment of Refractory Idiopathic Overactive Bladder Syndrome: A Retrospective Cohort Study

**DOI:** 10.3390/jcm12216783

**Published:** 2023-10-26

**Authors:** Janine Nicole Frey, Angela Vidal, Jörg Krebs, Corina Christmann

**Affiliations:** 1 Luzerner Kantonsspital Frauenklinik, Spitalstrasse, 6000 Luzern, Switzerland; vidalgutierrez.angelamaria@gmail.com (A.V.); corina.christmann@luks.ch (C.C.); 2 Clinical Trial Unit, Swiss Paraplegic Centre, 6207 Nottwil, Switzerland; joerg.krebs@paraplegie.ch

**Keywords:** refractory overactive bladder syndrome, percutaneous tibial nerve stimulation, treatment of overactive bladder syndrome

## Abstract

Background: Overactive bladder (OAB) is a syndrome defined as urinary urgency, accompanied by increased frequency and nocturia with or without urge incontinence, in the absence of urinary tract infection or other obvious pathology. The standard therapies are anticholinergic agents, selective beta-3 adrenoreceptor agonists, or intradetrusor injections of botulinum toxin (BTX-A). For patients with contraindications for BTX-A or drug therapies, percutaneous tibial nerve stimulation (PTNS) may be used. PTNS shows fewer side effects than anticholinergic drugs and costs less than BTX-A. The primary outcome of this study was to assess the efficacy of PTNS in women with refractory OAB. Methods: Women with refractory OAB undergoing PTNS at our tertiary referral center from 2017 to 2019 were included. The validated German Female Pelvic Floor Questionnaire and a micturition protocol were filled out before and after PTNS. PTNS was applied weekly for 12 weeks. Results: Improvements in OAB symptoms were seen in daily micturition frequency, urgency, and urgency incontinence from pre- to post-PTNS (*p* < 0.006). Impairments to quality of daily life were significantly (*p* < 0.0002) less severe after PTNS. There was a significant reduction in daytime voiding frequency from a median of nine to five (*p* < 0.0001). Conclusions: Substantial reductions in OAB symptoms, daily micturition frequency, urgency, and urgency incontinence were found in patients with refractory OAB after PTNS.

## 1. Introduction

Overactive bladder (OAB) syndrome is defined as urinary urgency, usually accompanied by increased frequency and nocturia with or without urge incontinence, in the absence of urinary tract infection (UTI) or other bladder-associated pathologies [1]. Idiopathic OAB is a chronic condition of urinary tract symptoms, of which the specific pathophysiology is yet unknown. In contrast, neurogenic OAB is a bladder dysfunction frequently seen in patients with multiple sclerosis, spinal cord injury, or Parkinson’s disease [2].

The prevalence of idiopathic OAB is approximately 16% in European and American adults and increases with age to up to 30% in women over 65 years [3]. It has a huge impact on quality of life, causing significant social, psychological, occupational, domestic, physical, and sexual problems [4].

Primary treatment options for idiopathic OAB include bladder retraining and uro-therapeutical interventions, including drinking assessments.

Second-line treatment options are based on pharmaceutical drug therapies, either with anticholinergic agents or a selective beta-3 adrenoreceptor agonist. Due to severe side effects, such as dry mouth, constipation, or cognitive impairment, women’s compliance with the use of anticholinergic agents is rather low [5,6]. Side effects, non-response, or monetary burden lead to the termination of drug therapy at a mean rate of 84% within 12 months [7,8].

Third-line modalities include the intravesical injection of botulinum toxin type-A (BTX-A), percutaneous tibial nerve stimulation, or the implantation of a sacral nerve stimulator (SNS). Improvements in symptoms after the injection of botulinum toxin are equivalent to those after anticholinergic therapy (60%). However, the most common side effects, such as elevated post-void residual volume, recurrent urinary tract infection, or repeat injections, are not always well tolerated by patients [9,10]. SNS is an important third-line treatment option, but it comes with certain drawbacks. One disadvantage is the requirement of a minimally invasive procedure to implant the device. Additionally, the device needs battery replacement every 5 to 10 years, depending on the specific model in use, which also entails another minimally invasive procedure. Moreover, ongoing lifelong follow-up with a urologist is essential for the maintenance of the device [11]. A long-term study of 325 patients with a mean follow-up of 7.1 years showed that up to 39% of the patients dropped out because of loss of follow-up, death, dementia, lack of efficacy, device problems, or infections [11,12]. Neuromodulation with percutaneous tibial nerve stimulation (PTNS) is an alternative treatment possibility. PTNS shows fewer side effects than anticholinergic drugs, costs less than BTX-A and SNS, and may, therefore, be a valuable option for treating OAB [3].

The posterior tibial nerve is a distal branch of the sciatic nerve, originating in the pelvis (L5-S3 spinal roots). Its stimulation leads to retrograde neuromodulation of the sacral segments S2/S3, which are part of the spinal center’s bladder control. There have been reports of clinically relevant effects, such as improving voiding frequency, nocturia, urgency, and incontinence episodes, as well as the quality of life in patients with OAB [3]. To date, two specific methods of neuromodulation have been discussed: the activation of the afferent fibers, which leads to reflex inhibition on a spinal or supraspinal level, or the activation of the efferent fibers to the striated urethral sphincter, reflexively inducing detrusor relaxation [2,13]. In PTNS, the posterior tibial nerve is stimulated through inserting a 34-gauge (0.16 mm) needle at a 60° angle, 4–5 cm cranial and 1 cm dorsal to the medial malleolus. The electrical current is a continuous square waveform with a duration of 200 μs and a frequency of 20 Hz. The intensity of the current is determined by the highest level tolerated by the patient [2,14].

## 2. Materials and Methods

The primary objective of this study was to assess the effectiveness of PTNS in women with refractory idiopathic OAB compared to first- and second-line therapies.

In this retrospective cohort, we included all women with refractory idiopathic OAB undergoing PTNS from 2017 to 2019 at the Cantonal Hospital of Lucerne, a tertiary referral center for urogynecology. All women had a full multichannel urodynamic assessment to identify the symptoms of idiopathic OAB. The Sedia^®^ Urodynamic Measurement System (year of production: 2018) served as the device for urodynamic measurements. The urodynamic studies included a conventional filling cystometry (with a maximal bladder filling of up to 500 mL) and a pressure flow study, according to the recommendations of the International Continence Society (ICS) [15]. Residual urine was measured utilizing clean intermittent catheterization. An increased post-void residual (PVR) volume was defined as ≥100 mL [1].

To differentiate concomitant stress urinary incontinence, clinical cough stress tests in the lithotomy and standing positions with a minimal bladder filling of 300 mL and/or pad weight tests were performed in order to assess overt SUI. Occult SUI (oSUI) was defined as clinically proven urinary leakage after prolapse reduction. A sponge holder was used to reduce the prolapse. The diagnostic follow-up was completed using a cystoscopy in order to exclude bladder pathologies.

Refractory OAB was defined as failed first-line treatment with either anticholinergic agents, selective beta-3 adrenoreceptor agonists, and/or BTX-A with no improvement or severe side effects. There were no patients with previous SNS treatment. All post-menopausal women were treated with a vaginal estrogen cream twice weekly. The exclusion criteria were ages under 18 years, neurological OAB, pregnancies during PTNS, or women unable to fill out the questionnaire and the micturition protocol due to language barriers or mental issues.

Data were obtained from patients’ gynecological hospital patient records. Demographics, comorbidities, and urodynamic parameters were collected. All women completed the validated German Female Pelvic Floor Questionnaire (GFPFQ) [16], which contains bladder, bowel, prolapse, and sexual function domains before and after PTNS. A greater score represents worse symptoms. A micturition protocol was completed before and after PTNS treatment. The completion of all the above-mentioned questionnaires and protocols is part of every urogynecological assessment at our clinic.

PTNS was applied unilaterally by a supervised nurse for 30 min once a week for 12 consecutive weeks, as described by A. Bhide et al. [17]. The posterior tibial nerve was stimulated through inserting a 34-gauge (0.16 mm) needle at a 60° angle, 4–5 cm cranial, and 1 cm dorsal to the medial malleolus. For stimulation, we used a low-voltage electrostimulator (Urgent^®^ PC Neuromodulation system, Cogentix Medical). Once the current (a continuous square waveform with a duration of 200 μs and a frequency of 20 Hz) was applied, flexion of the big toe, movement of the other toes, or a tingling sensation in the sole of the foot confirmed the correct positioning of the needle. The intensity (0–9 mA, adjustable in 19 steps) was always the highest tolerable level.

The subjective success rate was evaluated utilizing the GFPFQ before and after PTNS. The score entailing the bladder symptoms in the GFPFQ was taken for all patients, with a maximum overall bladder score of 45 points. A subgroup was assessed using the GFPFQ’s individual OAB-related bladder questions (items 1, 4, and 5) and questions relating to quality of life (items 14 and 15).

The objective improvement in OAB symptoms was evaluated using the micturition protocol before and after PTNS, documenting voiding frequency, including nocturia and voiding volume.

All patients signed general consent to share their data anonymously and informed consent for PTNS. Authorization by a cantonal ethics committee was obtained (EKZ 2019-01500).

The distribution of the data was evaluated using quantile–quantile plots and the Kolmogorov–Smirnov test. Data were reported as medians and quartiles or frequencies and percentages. The differences between the time points (pre- and post-PTNS) were tested using the Wilcoxon signed-rank test. The statistical analyses were performed using SPSS software (Version 25, IBM, Somers, NY, USA). A *p*-value of ≤ 0.05 was considered significant.

## 3. Results

The data of 48 women were analyzed. The GFPFQ’s individual OAB-related bladder questions were assessed in 39 women.

The median age at the beginning of the PTNS treatment was 66.5 years (54/74 years; range: 33–86 years). A total of 12 women (25%) had a BMI >30 kg/m^2^, 20 (42%) had urge incontinence, and 31 (66%) showed detrusor overactivity in the urodynamic tests (Table 1).

Overall, we observed a substantial and significant (*p* < 0.0001) reduction in subjective OAB symptoms after PTNS, with GFPFQ overall bladder scores decreasing from a median of 21 points (14.25/25) to 14 points (9/21).

**Figure 1:**
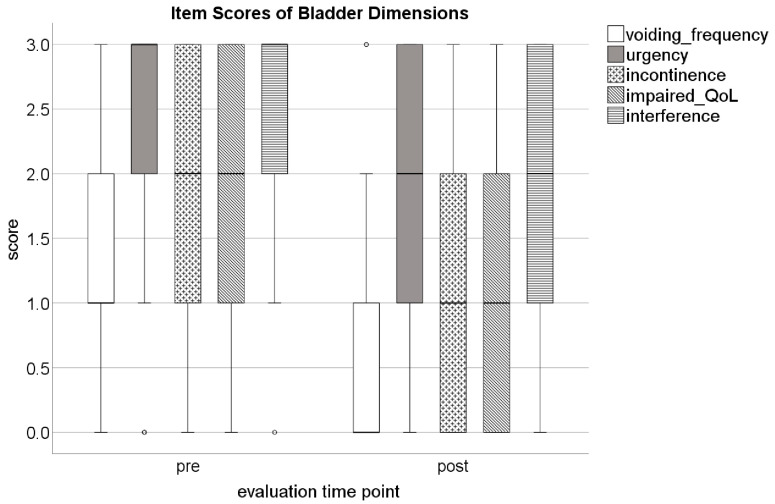
Box plots showing item scores of bladder dimensions before (pre) and after (post) percutaneous tibial nerve stimulation (PTNS). QoL: quality of life.

Specific subjective improvements in OAB symptoms were seen in daily micturition frequency, urgency, and urgency incontinence (items 1, 4, and 5 in the GFPFQ) from pre- to post-PTNS (*p* < 0.006). Both the impairment to the quality of daily life and interference by bladder problems (items 14 and 15 in the GFPFQ) were significantly (*p* < 0.0002) less severe after PTNS (Figure 1).

There was a significant reduction in the day- and nighttime voiding frequencies, from a median of nine (7.3/10) to five (4/6.75) (*p* < 0.0001) and one (0.6/2.75) to one (0/2) (*p* = 0.015), respectively.

No major complications related to PTNS were reported.

## 4. Discussion

This retrospective cohort study demonstrates that women with refractory idiopathic OAB symptoms benefit from third-line therapy with PTNS. In our cohort, PTNS caused a significant reduction in OAB-related symptoms. Significant improvements in the women’s quality of life were observed. The median voiding frequency (day- and nighttime) showed a significant reduction after PTNS, almost halving the number of daytime voidings.

A standardized stimulation protocol exists as far as where to place the needle and the electrical current form and the frequency used, as well as the stimulation time. However, some studies use unilateral application and some bilateral application. To our knowledge, there are no randomized studies comparing bilateral with unilateral PTNS application for improving OAB symptoms. No defined treatment protocol exists for the time of application. The regimens are heterogeneous and vary from 6 to 12 weeks, either with or without maintenance treatment. Maintenance treatment continued for up to 14 months, with mostly one PTNS session every 3 or 4 weeks [17,18].

Studies on PTNS, including RCTs and meta-analysis, have shown a significant effect on idiopathic OAB. For instance, a meta-analysis by Wang et al. [19] included 28 studies with 2461 patients showing a significant clinical effect on the voiding frequency per day, urgency episodes per day, and incontinence episodes per day. However, it is still unclear which patients with idiopathic OAB profit the most. Reviews of urodynamic data in 90 OAB patients treated with PTNS showed a better response in patients without detrusor overactivity [17,18]. Iyer S et al. [20] retrospectively reviewed 183 patients with refractory OAB who were treated with PTNS, including all women who had at least one PTNS session. They only found that the number of sessions was a success predictor but did not identify how many patients had urodynamics. Overall, urodynamic data on idiopathic OAB patients treated with PTNS are rare.

PTNS for refractory OAB is not well investigated. Similar to Iyer et al., Lashin et al. [21] conducted a PTNS-versus-sham study in a 6-week protocol on refractory OAB, stating a statistically significant improvement in bladder symptoms. Nevertheless, there were only 25 patients, both men and women, included for the PTNS treatment.

Furthermore, there is no clear evidence of the long-term efficacy of treating idiopathic OAB patients with PTNS. As discussed above, the protocol for the length of the PTNS sessions is very heterogeneous, and it is unclear whether patients benefit from additional maintenance PTNS treatment after the initial PTNS therapy. Studies such as the OrBIT trial [22] or that of Peters et al. [23] are small but have shown sustained improvement and have reported long-term efficacy and safety.

In real life, hospital-based PTNS therapy seems to be rather unpractical for longer periods. For patients, it is usually an inconvenience to travel weekly to a clinic for their PTNS session for more than 12 weeks of therapy. This often leads to poor compliance. Fortunately, there is also the possibility of the application of transcutaneous tibial nerve stimulation (TTNS). It can be self-applied at home using a surface electrode instead of a needle to stimulate the tibial nerve. Studies have shown that TTNS is an effective treatment option in idiopathic OAB patients [24,25,26]. In our cohort, we offered patients maintenance therapy with TTNS but did not evaluate this treatment.

The limitations of our study are, firstly, the retrospective observational design; secondly, the lack of detailed baseline information on comorbidities; and thirdly, the small number of patients. The fourth limitation is the short follow-up of only 6 weeks after completing 12 weeks of PTNS, so there is no evidence of whether a 12-week treatment of PTNS has a long-lasting effect. PTNS is an outpatient hospital-bound treatment, and there seems to be a need to further investigate the use of TTNS at home for patients after a successful PTNS treatment.

In addition to these limitations, the effectiveness of PTNS in our study was assessed through comparing symptom severity before and after treatment without a control group. Thus, we cannot exclude the possibility that the observed improvements were due to a regression to the mean. However, we included only refractory OAB patients whose previous medication treatment showed no effect or was discontinued due to side effects, indicating a benefit from PTNS.

## 5. Conclusions

In conclusion, a substantial reduction in subjective OAB symptoms, including daily micturition frequency, urgency, and urgency incontinence, was found in patients with refractory idiopathic OAB after the use of PTNS, which may indicate relevant benefits of this treatment with a very low risk of severe side effects, especially compared to first-line treatments.

However, to confirm the beneficial indications of our study, a randomized controlled trial of PTNS in refractory OAB, including its long-term effect, is needed, especially a study assessing two refractory idiopathic OAB cohorts, with or without detrusor overactivity, for PTNS versus PTNS plus drug therapies.

## Figures and Tables

**Table 1:** Patients’ characteristics.

	*n* (%)
BMI > 30 kg/m^2^	12 (25)
Urge only	37 (77)
Urge incontinence	20 (42)
Detrusor instability in urodynamic testing	31 (66)
Previous treatment with anticholinergics	23 (48)
Previous treatment with selective beta-3 adrenoreceptor agonists	20 (42)
Previous treatment with anticholinergics and selective beta-3 adrenoreceptor agonists combined	5 (10)

## Data Availability

For data supporting the reported results, please consult with the corresponding author.
